# Sargassum seaweed in the Caribbean: A major public health problem still unsolved

**DOI:** 10.7189/jogh.13.03017

**Published:** 2023-03-17

**Authors:** Dabor Resiere, Hatem Kallel, Jonathan Florentin, Rishika Banydeen, Keats Compton, Papa Gueye, Hossein Mehdaoui, Remi Neviere

**Affiliations:** 1 Department of Critical Care Medicine, University Hospital of Martinique, Fort-de-France, Martinique, France; 2 Department of Critical Care Medicine, General Hospital of Cayenne, Cayenne, France;; 3 Department of Clinical Research, CHU Martinique (University Hospital of Martinique), Fort-de-France, France; 4 Department of Cardiology, CHU Martinique (University Hospital of Martinique), Fort-de-France, France

**Figure:**
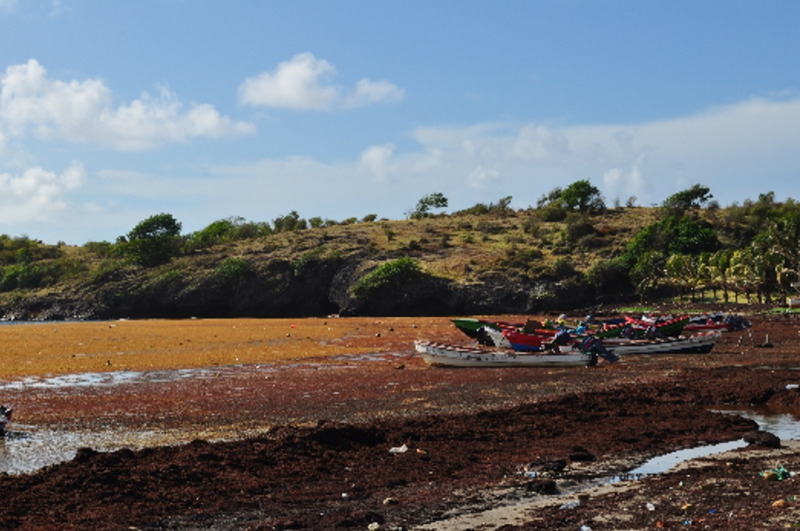
Photo: Fishing village of Praslin, Saint-Lucia east (Atlantic) coast. Source: personal collection of Keats Compton, a member of the group. He has provided consent to publish.

For the past decade (beginning in 2011), excessive quantities of sargassum have been accumulating not only in the Caribbean Sea (Photo) but in Central America, North America, and Africa [1-3]. During this period, practically all governmental focus in the Caribbean has been directed mainly on coastline cleanup (as a response to pressure from fishing communities whose vessels were often marooned) and agro-processing of the weed into fertilizer or landfills. Fishing communities tend to be concentrated in rural areas and make significant contributions to the local economies and can jointly command governments’ attention, as with the focus on clearance for marooned boats. Governments would not, however, help with the repair of damaged engines.

By comparison, the health hazards posed by decomposing sargassum have not commanded the same attention from governments as of yet. Regional “seminars/workshops” on sargassum have usually addressed the logistics of coping with the influx, admittedly with little actual follow-up. The United Nations Secretary-General even visited a site during his attendance at the 40th CARICOM Heads of Government meeting in 2019 in St. Lucia. However, this has been recognized as nothing more than a photo-op provide by an important locally staged event. The local authorities failed to take advantage of such an important visitor to give international recognition to the sargassum phenomenon in the Caribbean.

To date, nothing has really been done in the health sector to address the management of the released hydrogen sulfide and ammonia, or to ascertain the scale of the toxins released during the decomposition of the sargassum. It remains unclear as to just how these health hazards can be placed on the agendas of regional organizations.

Decomposing sargassum releases hydrogen sulfide gas and ammonia, which can cause respiratory, skin, and neurocognitive symptoms in both local residents and tourists. Toxic exposure typically happens during decomposition, approximately 48 hours after it washes ashore. Significant exposures (50-400 ppm) may produce difficulty in breathing, agitation, confusion, nausea and vomiting, elevated blood pressure, and loss of consciousness. At higher concentrations, H2S rapidly causes myocardial infarction, unconsciousness, seizures, acidosis, and death [4]. Hydrogen sulfide is absorbed by the upper respiratory tract mucosa and causes histotoxic hypoxemia and respiratory depression by exerting an inhibitory effect on cytochrome oxidase A3 [5]. There is no evidence for specific treatment, as even a large dose of Hydroxocobalamin does not seem to rely on the trapping of H2S.

Caribbean governments are aware of the adverse impact of sargassum standings on their hotel and tourism sectors. About 70% of the territories derived an average of 20% of GDP from this sector in 2019 [6]. Governments could be expected to downplay the sargassum invasion to protect that 20% contribution. The COVID-19 pandemic and the closure of borders negatively impacted the countries’ economies. Restarting the tourism industry will be a high priority as it makes up more than 70% of the countries’ economies. This will also mean further downplaying of the sargassum invasion.

The advent of the COVID-19 pandemic produced a major shift in the focus of regional governments in the area of health care provision and management. The gravity of the pandemic has resulted in already stretched health care systems being overwhelmed. As of October 2021, infections and deaths continue to rise; countries are forced to devote resources to COVID-19 disproportionately, and consequently, routine demands on health services are unable to be satisfied.

In this climate, and considering that the health hazards of sargassum are yet largely undetermined, it is inevitable that the sargassum health risk(s) will not get the warranted attention for the time being. Whereas island governments might be able to postpone addressing the health hazards, they have been subjected to pressure from their fishing communities, whose boats, apart from being ocassionaly marooned inshore as stated above, have seen the electronics in their increasingly sophisticated outboard engines affected by the gases emitted during decomposition, as well as in some domestic appliances. This pressure has resulted in government-funded coastal cleanups, albeit sporadically.

To date, the Caribbean islands have not come together to address the common problem of sargassum stranding, for reasons which remain unclear. The same can be said of the approach to the COVID-19 pandemic. The time may well have come for medical professionals in the region to convince policymakers of the need to promote a coordinated approach to tackle common health problems. As a pre-requisite, awareness of potential health hazards will need to be created.

In the French territories of America (Guadeloupe & Martinique), the situation remained unchanged. Despite the French Government’s plans to tackle the sargassum problem, these toxic algae are continuing to inundate the coasts of Martinique, Guadeloupe, and French Guiana in ever-greater volumes [7].

In 2018, the former French minister for the environment presented a €10 million plan to combat the problem, but people are still living and working in the affected regions. However, a total amount of €13 million has been reallocated by the funding partners from the French National Research Agency to tackle the problem. Last year, another sargassum seaweed plan was announced by the French government. The problem has also been raised in the European parliament last November.

Four years ago, we sounded the alarm by publishing our article on sargassum as a public health problem [2]. Nothing was done until last November, when the Territorial Assembly of Martinique, through the FEDER Agency, provided funds to finally initiate our research work – €1.2 million has been allocated to us.

Meanwhile, the public continues to be adversely affected, some have sold their dream houses which are becoming unlivable, some have abandoned their schools and workplaces for lack of a solution to this scourge. It is urgent to come to the aid of these families who, in addition to the health consequences due to the significant emanations of hydrogen sulfide, have to bear the material consequences, being often forced to replace all their household appliances or the metal parts of their houses.

Today, there is no national and international consensus on facing this public health problem. There is no Caribbean network or a broad consensus to advance research at this level [8].

In conclusion, governments in the Caribbean must invest more in sargassum management. Within countries, more collaboration across agencies and sectors (government, Caribbean Public Health Agency (CARPHA), Organization of the Eastern Caribbean States (OECS), civil society, research, United Nations Educational, Scientific and Cultural Organization (UNESCO) and the Caribbean Community (CARICOM) is warranted. Collaboration across countries can enhance faster progress and reduce consequences and deaths due to sargassum.
